# Telemedicine in Otolaryngology in the COVID-19 Era: A Year Out

**DOI:** 10.7759/cureus.20794

**Published:** 2021-12-29

**Authors:** Jason F Ohlstein, Omar G Ahmed, Jordan Garner, Masayoshi Takashima

**Affiliations:** 1 Department of Otolaryngology - Head and Neck Surgery, St. Luke’s University Hospital, Bethlehem, USA; 2 Department of Otolaryngology - Head and Neck Surgery, Houston Methodist Hospital, Houston, USA

**Keywords:** covid-19, virtual clinic, tele health, virtual consultations, telemedicine (tm)

## Abstract

One year ago, shortly after the onset of the coronavirus disease 2019 (COVID-19) pandemic, we published our initial experience with telemedicine. We showed that during the early pandemic, there was a dramatic shift to telemedicine and that 70% of our patients would decline telemedicine in favor of an in-person visit. As clinical limitations and stay-at-home orders relaxed, we sought to define how we have used telemedicine since. After the initial month of the pandemic, our utilization of telemedicine fell to an average of only 5% of visits over the past year. Nearly 80% of all telemedicine visits were routine follow-up visits, with its usage being unaffected by local policy and pandemic surges. The usefulness and applications of telemedicine have been well described; however, after our initial reliance on telemedicine, its use has been minimal. Moving forward, attention will need to focus on innovation and expanding comprehensive virtual examinations for otolaryngology to fully embrace this technology.

## Introduction

There has been a rapid acceleration in the use of telemedicine as a result of the coronavirus disease 2019 (COVID-19) pandemic. We previously described our initial experience with telemedicine at a tertiary otolaryngology academic clinic during the onset of the COVID-19 pandemic [1]. We showed that the majority of our patients would decline a virtual visit due to the perceived lack of a physical exam. We now look back at our experience with telemedicine and the evolving role it has played in our everyday practice over the past year, as well as the impact of changing policy, mask mandates, surges, vaccines, and emerging new variants.

## Materials and methods

Between March 2020 and July 2021, total patient visits to our tertiary referral center in downtown Houston, Texas were accessed and dichotomized into visits conducted virtually and those which were held in person. Percent in-person vs. virtual visits was then calculated. Visits were only considered for our physicians with physician extenders and other nursing visits were excluded from the analysis. Data pulled from the Houston Methodist Hospital system for monthly new inpatient COVID-19 positive admits were then used as a surrogate marker for local COVID-19 severity. Additional subgroup analysis was performed to analyze for any differences between our subspecialty departments. Additionally, key policy dates were gathered, which included relaxation of masking and surgical restrictions. Prism 8 (GraphPad Software, San Diego, CA) was used for all analysis and data preparation. This study met all institutional and IRB requirements.

## Results

Our initial study showed during the first two months of the pandemic that 72% of our patients declined a virtual visit, primarily for the reason that they desired a physical exam. We additionally demonstrated that patients with facial plastic visits were more likely to accept a virtual visit, and those with otology visits were more likely to decline [1]. To put this study in a temporal context, it captured a moment in time defined by stay-at-home orders and uncertainty with the pandemic and was prior to loosening of clinical restrictions. As such, we sought to answer the question: what role has telemedicine been playing since then?

**Figure 1:**
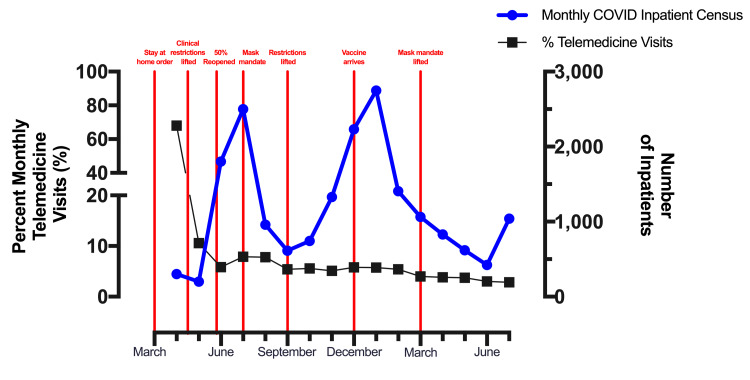
Monthly telemedicine visits compared to local pandemic severity Percent total monthly telemedicine visits. Additionally, plotted is the monthly total of new inpatient COVID-19 admissions as a surrogate for the local severity of the pandemic. Several key policy changes are marked along the x-axis.

To answer this question, we reviewed the percentage of our total monthly visits that were virtual over the past year. To put these data into the further context of the pandemic and local policy, we compared this percentage to the monthly inpatient COVID-19 census, used as a surrogate marker for local severity of the pandemic and important policy changes (Figure 1). We found a drastic decrease in the utilization of telemedicine from 68% of all monthly visits in April to a level of 5% for the remainder of the study with the relaxation of clinical restrictions in mid-April 2020. Telemedicine utilization was largely unaffected by pandemic severity and local policy with the exception of a slight transient increase in the utilization of virtual visits during July and August 2020 from 5% to 8%, which coincided with the implementation of a state-wide mask mandate during a COVID-19 peak.

We looked to see how our different subspecialties utilized telemedicine and found that, on average, over the past year, our facial plastics providers had the highest usage. Of all facial plastic patient visits, 14% were telemedicine, compared to just 5% with our otology providers and an average of 9% for the remaining specialties. When looking at the totality of how telemedicine was utilized by our physicians, it was mainly used for follow-up visits, new visits only accounting for 21% of all visits. Suggesting that telemedicine was most useful for routine follow-up, review imaging, or assessing subjective response to treatment.

## Discussion

While an average of 5% of all monthly visits being virtual has increased from pre-pandemic levels, telemedicine still accounts for a small portion of our clinical practice. There is no doubt that telemedicine has become a feasible means of delivering care in our field [1-4]. With greater use over the past year, billing and reimbursement for telemedicine has become more standardized and offer similar reimbursement to in-person encounters, raising its appeal for some clinicians [5-7]. From an institutional standpoint, there was no pressure to use or not use telemedicine in our practice with its utilization left up to the provider and patient demand. So, the question remains: why are otolaryngology patients and providers not utilizing telemedicine?

There are several potential applications to telemedicine in our field, including increasing access to care for the underserved and those in rural areas. Despite studies showing high levels of both physician and patient satisfaction with telemedicine, otolaryngology has been slow to adapt. Our slow adoption of virtual medicine has been attributed to our routine use of technology, such as endoscopy, and our concomitant use of ancillary services such as audiology [4,8]. As shown in our first study, most patients would decline a virtual visit for the sole reason that they are not getting an in-person examination. Perhaps this preference lies in the fact that most patients are referred to us by another provider, and there is an expectation of an advanced level of evaluation. On the other hand, we as providers are also accustomed to using all of our tools for evaluation. There is an increasing need for the development of virtual examination tools that will allow otolaryngologists to better assess and diagnose pathologies. Until we have better ways to examine a patient virtually, there will be some reluctance for telemedicine in our field. The future of telemedicine in otolaryngology will be largely driven by advancements in technology that will allow both the patients and clinicians to feel comfortable carrying out a comprehensive patient visit.

## Conclusions

Over the past year, telemedicine has proven to be a rapidly scalable lifeline and a convenient means of delivering care for many specialties. Despite our initial reliance on telemedicine, with the relaxation of clinical restrictions, the use of telemedicine drastically decreased and was largely unaffected by local policy and COVID-19 levels. Telemedicine in otolaryngology is still lacking in providing a pertinent examination that otolaryngologists need. Telemedicine will need to continue to innovate for our specialty to embrace this technology.
